# A Robust Facial Expression Recognition Algorithm Based on Multi-Rate Feature Fusion Scheme

**DOI:** 10.3390/s21216954

**Published:** 2021-10-20

**Authors:** Seo-Jeon Park, Byung-Gyu Kim, Naveen Chilamkurti

**Affiliations:** 1 Department of IT Engineering, Sookmyung Women’s University, 100 Chungpa-ro 47 gil, Yongsna-gu, Seoul 04310, Korea; sj.park@ivpl.sookmyung.ac.kr; 2 La Trobe Cybersecurity Research Hub, La Trobe University, Melbourne, VIC 3086, Australia; N.Chilamkurti@latrobe.edu.au

**Keywords:** deep learning, facial expression recognition (FER), 3D convolutional neural network (3D CNN), multirate signal processing, minimum overlapped frame structure, self-attention, multi-depth network

## Abstract

In recent years, the importance of catching humans’ emotions grows larger as the artificial intelligence (AI) field is being developed. Facial expression recognition (FER) is a part of understanding the emotion of humans through facial expressions. We proposed a robust multi-depth network that can efficiently classify the facial expression through feeding various and reinforced features. We designed the inputs for the multi-depth network as minimum overlapped frames so as to provide more spatio-temporal information to the designed multi-depth network. To utilize a structure of a multi-depth network, a multirate-based 3D convolutional neural network (CNN) based on a multirate signal processing scheme was suggested. In addition, we made the input images to be normalized adaptively based on the intensity of the given image and reinforced the output features from all depth networks by the self-attention module. Then, we concatenated the reinforced features and classified the expression by a joint fusion classifier. Through the proposed algorithm, for the CK+ database, the result of the proposed scheme showed a comparable accuracy of 96.23%. For the MMI and the GEMEP-FERA databases, it outperformed other state-of-the-art models with accuracies of 96.69% and 99.79%. For the AFEW database, which is known as one in a very wild environment, the proposed algorithm achieved an accuracy of 31.02%.

## 1. Introduction

Communication skills have been developed based on the senses that play an important role in human interaction. There are five human senses: sight, sound, touch, taste, and smell. There is no doubt that sight is the most important one of the five senses for most people, since up to 80% of all senses are recognized through sight [1].

In recent years, the importance of human–computer interaction (HCI) grows larger as the artificial intelligence (AI) field develops. The basic goal of the HCI field is to improve the interaction between human and computer systems by making the computers more useful and accessible to humans. Additionally, the ultimate goal of the AI technology is to allow the machine to catch the user’s intentions or emotions by itself, thereby reducing the burden of the user and making it more enjoyable. Therefore, understanding the feelings and the action of the human becomes important in various human-centric services. This technology based on the human face is called facial expression recognition (FER) technology.

FER technology has many applications in customer service [2], the automotive industry [3], entertainment [4], and home appliances [5]. There are good examples including: games with different modes based on classifications of the user’s facial expression [6], identifying the driver’s drowsiness and instructing an appropriate response [7,8], automatically collecting vast amounts of data necessary for the study of human emotional behavior patterns [9], detecting the emotional state of the patient and predicting the situation in need of help [10,11], and establishing an adaptive learning guidance strategy by grasping a student’s psychological state using facial expressions and words that are used [12,13,14]. In recent years, interest and research on the development of intelligent home appliances and software that respond to the user’s emotional state have been focused on.

One of the main technologies for emotion recognition is to recognize a user’s emotional state from facial expression from an image sensor. Among various fields of biometrics, the face is a very important element that can be easily encountered in daily life. The emotional state that appears in the facial emotions is sufficient to be used as a human interface when sharing opinions with other people in the process of communicating with each other or conveying one’s feelings. Reflecting this importance, many studies related to FER have been conducted. In the field of psychology, many studies on facial analysis and recognition have been done for many years.

According to a study by psychologist Ekman and Friesen, six emotions of a person, happiness, sadness, anger, surprise, disgust, and fear, have been classified as basic emotions that are perceived in common without being influenced by each culture [15,16]. Based on this, many studies have classified six emotions or seven emotions adding neutral expressions to identify emotional states. In recent years, research targets are expanding to expressions including not only depression, pain, and sleepiness but also expressions representing mental states such as agreement, concentration, interest, thinking, and confusion. In addition, research is also being conducted on the recognition of natural facial expressions and not only the research through an ideal database containing exaggerated expressions to the limited environment. However, despite these efforts, FER technology is still at a level that can be applied only in limited circumstances.

The FER system that recognizes facial expressions consists of three steps. The first step is to detect a person’s face. This step is to detect a face area from an input image and to detect face elements such as the eyes, nose, or mouth. Representative algorithms include Adaboost [17], Haar-cascade [18,19], and the histogram of oriented gradients (HOG) [20]. Second, facial features are extracted from the recognized face using a geometric feature-based method or an appearance feature-based method. Finally, there is a classification step in which emotions are classified using the method based on the extracted features.

Facial expression recognition is a field with high dependency on datasets. There are two types of factors that influence facial expression recognition. The first type of external factors is uniqueness of each person such as gender, race, and age. The second type of external factors is the environment such as lighting, poses, resolution, and noise. However, many facial-expression datasets were created in controlled environments, so the second type of external factors were affected less than the first type. To overcome this problem, the dataset must be rich enough to accommodate these factors. Therefore, we used data augmentation to supply various information. Another method is to create a cross dataset that uses multiple datasets. This is to learn and test by combining different datasets under the same conditions. Through this, there is an advantage that facial expressions in a more diverse environment can be generalized.

Datasets used for FER are largely divided into two types according to the type of dataset. A static dataset consists of static images, and a dynamic dataset consists of dynamic images, which are called videos. In order to apply the FER in practice, we need to use a dynamic dataset, which is found in real life. In general, the accuracy of a dynamic dataset is lower than a static dataset, because dynamic images have different features such as facial movements over time. Therefore, temporal dynamics must be considered. Through the 2D convolutional neural network (2D CNN) [21], only spatial features can be identified within an image. Therefore, to classify the facial expressions in dynamic images through this 2D CNN, there is a limitation in processing temporal motion.

To solve this problem, a network dealing with the time axis is needed. Recurrent neural networks (RNN) are a type of artificial neural network that forms a circular structure in which hidden nodes are connected by directional edges. Data appearing sequentially can be usefully processed through RNN [22]. However, if the distance between the relevant information and the point where the information is used is long, the gradient gradually decreases during back-propagation, leading to a problem that the learning ability is greatly degraded. This is called the vanishing gradient problem. The long short-term memory (LSTM) [23] was devised to overcome this problem. LSTM is a structure in which the cell state is added to the hidden state of the RNN. Another method is the use of 3-dimensional convolution neural networks (3D CNN) [24]. Unlike conventional 2D CNN, 3D CNN uses a 3D convolution kernel to extract features not only for the space domain but also for the time domain.

In [25,26], they used geometric features such as landmarks, and the reference of a facial expression such as neutral expression was required while extracting features. However, in the case of real-life FER, no reference is given, and it cannot be guaranteed that the face of the neutral expression will be given. Therefore, a model that can recognize facial expressions without a reference is needed to use FER in practice.

We proposed a new facial expression recognition model to solve these problems. First, a 3D CNN structure that can simultaneously extract spatial and temporal features was used to obtain more accurate facial expression recognition results. Second, we used multi-networks with different frame rates to extract various features. The frames used for inputs entering each network should not overlap as much as possible, so we can utilize more spatio-temporal information. Third, we applied self-attention to the features that were extracted from each network, to make more reinforced features. The facial expressions were classified by combining these features through a joint fusion classifier.

In order to make a facial expression recognition model, the most relevant contributions are as follows:

- We defines a multi-depth network based on multi-frame rate input.
- A structure that minimizes the overlapping between input frames to each model was designed.
- The proposed scheme reinforced the features that are the result of the networks by self-attention, and it showed a better result than each network’s result.
- We verified the robustness of the multi-depth network on the variation of dataset and different facial expression acquisition conditions.

The rest of the article is organized as follows. The related works for facial expression recognition are introduced in Section 2. Section 3 introduces a detailed description of the facial expression recognition algorithm composed of five main steps. Section 4 provides several experimental results and the performance comparison results with the latest models. Finally, the concluding remarks of this article are given in Section 5.

## 2. Related Works

### 2.1. The Facial Expression Recognition Methods

#### 2.1.1. Classical Feature-Based Approaches

Features representing facial expressions are divided into the permanent facial features (PFF), which expresses permanent facial features such as the eyes and nose, and the transient facial features (TFF), which expresses wrinkles or protrusions that occur temporarily as facial muscles move [27]. In face recognition, the proportion of the PFF is large, but in the field of facial expression recognition, the TFF also plays an important role as well as the PFF. Representative methods of expressing these facial features in an image include a geometric feature-based method and an appearance feature-based method. Analyzing the existing studies in terms of expressing facial features is as follows:

##### Geometric Feature-Based Method

Systems based on geometric features express changes in the shape and expression of a face by using the positions of various facial elements and the relationships between them. Since the positions and movements of the mechanical features of the face are changed according to the difference between the shape of the face and the facial expression, an intuitive expression recognition method can be used by using dynamic information obtained by tracking these features from a video image. The difficult point of the geometric feature-based method is that because each person has a different face shape, the location of the feature cannot be used as it is. To solve this problem, the facial parts are modeled with the active appearance model (AAM) [28] or action unit (AU) [29] according to facial expressions, and based on the information extracted from the image, they are tracked to obtain the relative distance between the parts.

The geometric feature has the advantage of being able to implement a system that requires less memory and can easily adapt to changes in the position, size, and orientation of the face because the motion of the feature can be simply expressed with a few factors. On the other hand, since it is difficult to express the TFF that appears temporarily while the expression occurs, the geometrical features are similar, but there is a limit to distinguishing expressions with different facial textures such as wrinkles.

##### Appearance Feature-Based Method

The facial expression recognition method based on appearance features can accommodate both permanent features such as the eyes and mouth according to facial expressions and temporary features such as wrinkles for the entire image or the regional image. The appearance feature-based method is divided into a holistic image-based approach and a local image-based approach according to the size of the image used for feature extraction.

**Holistic Feature-Based Method**   The holistic feature-based method considers each pixel constituting a face image as one feature element and expresses the entire image as one feature vector. Therefore, when the number of pixels constituting the face image is large, the size of the feature vector becomes excessively large, and the amount of calculation increases accordingly. As a solution to this problem, the linear subspace method (LSM) was proposed. LSM [30] improved the overall processing speed and accuracy by expressing the feature vector composed of the pixels of the face image as a low-dimensional spatial vector through linear transformation. Representative LSMs include principal component analysis (PCA) [31], linear discriminant analysis (LDA) [32], and independent component analysis (ICA) [33].

This holistic feature-based method is simple because it targets the entire image without going through a separate feature extraction process, but it has a disadvantage in that its performance is poor in a dynamic environment in which the face pose, lighting, and facial expressions move.

**Local Feature-Based Method**   The regional feature-based method constructs a feature vector representing the overall face shape by setting a regional window in a region where changes can occur due to facial expressions in a face image and extracting features based on the brightness distribution within the window. In general, since the lighting of an image or changes in facial expressions appear in a part of the facial image, the regional feature-based method sets a local window only in the area where changes in the face can occur. Therefore, it has the advantage of being relatively less sensitive to these changes compared to the global feature-based method. Representative methods based on regional features include the Gabor filter [34], the Haar-like feature [18], and the local binary pattern (LBP) [35].

#### 2.1.2. Deep-Learning-Based Approaches

Most facial expression recognition algorithms used in recent studies use deep learning-based methods. When AlexNet showed a performance improvement in the ImageNet challenge [36], many researchers began to apply the 2D CNN structure to various fields, and it was also applied to the FER [37,38]. There have been many attempts to apply 2D CNN to the video frames. However, 2D CNN has structural limitations because they cannot provide temporal information to the neural network.

Many studies use two architectures to overcome this problem. First, 3D CNN was designed by transforming the structure of 2D CNN [24]. 3D CNN uses a 3D convolution operation, which has three-dimensional convolution filters. Therefore, the feature map generated by one filter is also three-dimensional, and 3D CNN can learn temporal learning of successive frames from the convolution filter. This structure enabled spatio-temporal-feature learning for short-term input frames. Second, a hybrid method that combines multiple networks was also used. A CNN-RNN or CNN-LSTM [39,40] structure is one of the examples. It learns spatial features with CNN and then learns temporal features by RNN or LSTM.

A hybrid method is also used for improving accuracy as well as solving 2D CNN problems. In [26,41], they used two networks to extract temporal appearance and geometric features from image sequences and facial landmark points. They combined these two networks with a new integration method to make the two models cooperate with each other and improve the performance. Based on these methods, a hybrid method that combines multiple-depth networks based on 3D CNN is suggested.

### 2.2. Multirate Filter Bank

In [42], multi-rate filter banks produced multiple output signals by filtering and sub-sampling a single input signal or, conversely, generating a single output by up-sampling and interpolating multiple inputs. An analysis filter bank divides the signal into *M*-filtered and sub-sampled versions. A synthesis filter bank generates a single signal from *M*-up-sampled and interpolated signals. The proposed algorithm looks like a sub-band coder, which was combined by an analysis filter bank and a synthesis filter bank.

We divided the input video (dynamic image) into multiple outputs, which have different frame rates, and put them into networks, which have different network-depth models. By using this structure, we could construct various spatio-temporal features. These features were combined into one feature, and we classified it by a joint fusion classifier.

### 2.3. Self-Attention

Attention is a methodology that started from the perspective of “let the model learn even the parts that need to be learned intensively for better performance.” It makes network-to-weight features and uses the weighted features to help achieve the task. It is widely used in natural language processing (NLP), multivariate time series, and machine translation.

The attention mechanism was first devised for sequence learning [43]. It figures out which output sequence of the encoder is most associated with the particular output sequence of the decoder.

The attention itself is almost similar to the transformer [44]. The transformer can be divided into self-supervision and self-attention. By self-supervision, it is possible to train a model with an unlabeled dataset and learn generalizable representations. Self-attention calculates the attention by itself, and it assumes a minimum inductive bias unlike models such as CNN and RNN.

**Figure 1:**
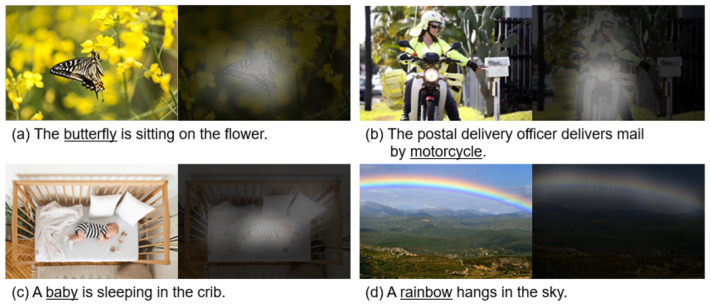
The examples of attending to the correct object (*white* indicates the attended regions, and *underlining* indicates the corresponding objects).

The self-attention method has been applied in computer vision tasks such as [45,46,47,48,49]. In [45], they inserted an attention block between convolutional layers to improve image feature creation performance. In [46], the attention was performed per channel through a dot product on the channel characteristic vector, and the authors used a channel and spatial attention block in [47]. Figure 1 shows some examples of the visual attention.

## 3. Proposed Scheme

This section introduces the proposed method in detail. Section 3.1 introduces the method of how we pre-processed the input images before feeding them into the networks. Additionally, we describe the data augmentation process in Section 3.2. Section 3.3 elaborates the network that was used to extract the feature maps. Section 3.4 goes into detail about how to reinforce the features and the joint fusion classifier, which classifies the facial expressions with the reinforced features.

**Figure 2:**
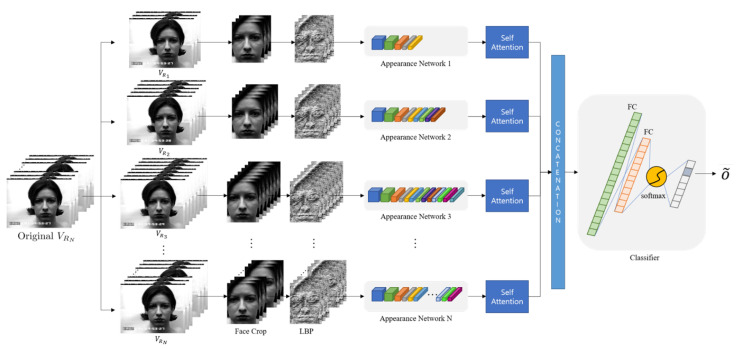
The process of the proposed facial expression recognition scheme.

Figure 2 shows the overall structure of the proposed algorithm based on multirate inputs and multi-depth networks to make a robust scheme.

### 3.1. Data Pre-Processing

**Figure 3:**
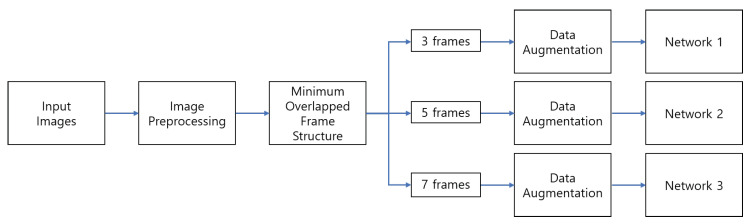
The entire process of making input dataset with a sequence.

The environments of each database such as resolution, brightness, and pose are changeable. In order to have a general environment, a data pre-processing step is required, and Figure 3 shows the entire process of input with one sequence.

We augmented the pre-processed dataset to avoid the overfitting problem. Then, each network received those dataset as input since CNN requires the fixed size of the input. Through this process, unnecessary sequence parts were removed, and important features were highlighted, so that the network can extract informative features efficiently.

#### 3.1.1. Image Pre-Processing

**Figure 4:**
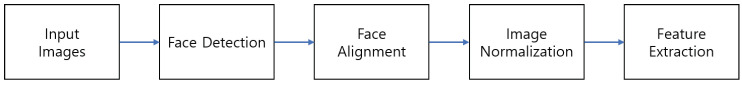
Architecture of data pre-processing algorithm.

In order to have a general condition of the input, we went through four steps. The flowchart of the image pre-processing algorithm is shown in Figure 4.

##### 3.1.1.1. Face Detection

For FER, we needed to detect the face area first. Then, we cropped the detected face area not to be affected by unnecessary parts such as hair or accessories.

We used the FaceBoxes module [50] to detect the face region. It consists of the rapidly digested convolution layers (RDCL), the multiple scale convolution layers (MSCL), and the divide and conquer Head (DCH).

##### 3.1.1.2. Face Alignment

**Figure 5:**
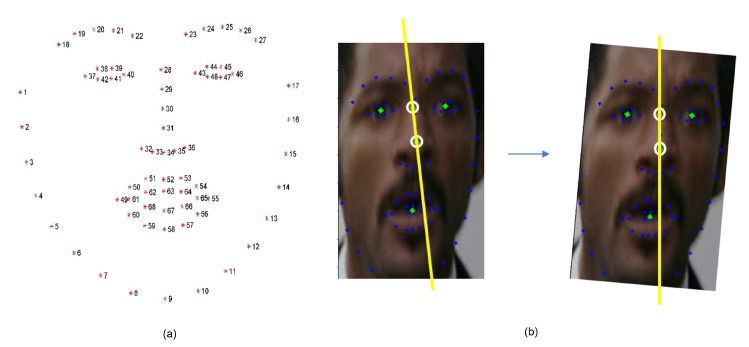
Aligning the face: (**a**) 68 facial landmarks; (**b**) face alignment with the landmarks.

Through facial landmarks, we checked whether the face is frontal or not and aligned the askew frontal face in order to fix the posture. We used the style-aggregated network (SAN) module [51] to extract the landmark of the face. We tilted the face by aligning the *x* axis of the tip of the nose and the *x* axis of the center of the eyes vertically. The tip of the nose was the 34-th landmark, and the center of the eyes was the average of the 37-th to 46-th landmark—refer to Figure 5a.

After alignment, the face was judged to be front if the 34-th landmark, which is the tip of the nose, was between the 40-th landmark and the 43-th landmark, which are the nearest points from the nose of the left and right eye. The example of this part is shown in Figure 5b. After the face alignment process, we cropped the minimized face area without empty data again. Then, we resized the image into 128 × 128 in order to make the same resolution. This alignment process can be considered as a kind of affine transformation based on two points. This had two constraints as: (1) the images of the two lines were also parallel, and (2) translations are isometries.

##### 3.1.1.3. Image Normalization

There are two ways to normalize an image. The first is to normalize the size of the image. In general, when using CNN, the dimension of an input image or feature needs to be fixed. Therefore, we resized all the input images into the same size 128 × 128. This accelerates the convergence of the network. The second is to normalize the image numerically. It means we normalized the pixel distribution of the original image. Through Equation (1), which has been reported in [35], the values followed the standard normal distribution standardized by the Z-score. The standard conversion formula for this is as follows:

(1) $$x^{\prime }=\frac{x-\mu }{\sigma }.$$

Here, *x* is the pixel value of the original image, and $x^{\prime }$ is the new value of the converted image. In addition, μ is the average pixel value of the image through calculation, and σ is the pixel standard deviation value of the image through calculation. The data subjected to Z-score standardization showed a normal distribution with an average of 0 and a deviation of 1 approximately. This intensity normalization can give better features than using one by 255.

In most of the deep learning approaches, an input image is given into the designed deep neural network after normalizing it by 255, to make robustness in illumination change. However, it always gives an intensity range as (0, 1.0). That is, this normalization by 255 compresses into too small an intensity range. However, the suggested Z-score maintains a larger range as (−1.0, +1.0) by the standard deviation of illumination in the given image. Through experiment, we verified the suggested normalization to be more effective to make features in convolution neural networks.

##### 3.1.1.4. Feature Extraction Using LBP

We extracted features from the resized image to reduce the computational complexity and to emphasize facial characteristics. In this study, facial features were extracted through an LBP. The LBP classifies the texture of the image and is widely used in fields such as facial recognition and gender, race, and age classification [52,53]. Additionally, the LBP function was used to eliminate the effect of lighting.

In [54], Timo et al. proposed a method of applying LBP to facial recognition problems for the first time. This showed a better result than many of the existing approaches.

**Figure 6:**
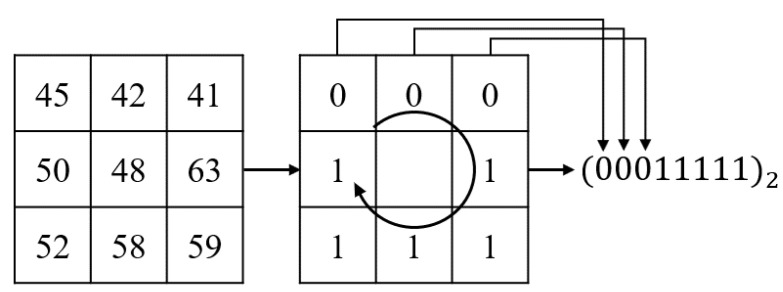
The LBP feature extraction.

In order to have the LBP feature value for one pixel, a 3 × 3 size block was used, and it is shown in Figure 6. Each pixel value except the center was compared with the pixel value located in the center, and if it was brighter than the center, it was encoded as 1; if it was darker than the center, it was encoded as 0. The formula is as follows:

(2) $$LBP\left(x,y\right)=\sum_{n=0}^{N-1}s\left(p_{o}-p_{c}\right)\times 2^{n},$$

where

(3) $$s\left(x\right)=\left\{\begin{array}{cc} 1, & \mathrm{if}\;x>0,\\ 0, & \mathrm{otherwise}. \end{array}\right.$$

The value of the center point s(x) was converted to a binary number 0 and 1 through Equation (3) where *x* refers to the difference between the center pixel $p_{c}$ and the other pixel $p_{o}$. As value of the center pixel $p_{c}$, a different 8-bit binary string was generated if *N* is 8. Then, the binary code was converted to decimal LBP(x,y) by Equation (2). The LBP’s capabilities help reduce computational complexity compared to the original image. It also emphasizes the main texture of the face in the image.

#### 3.1.2. Minimum Overlapped Frame Structure

The proposed model extracts features from multiple networks, whose inputs are various using different input frame rates, and classifies facial expression by combining extracted features. Therefore, we thought that it would be more efficient to learn if various information is given.

In the conventional structure, frames are extracted with regular intervals. This assumes that the expression of the sequence goes from the neutral to the peak. When the number of the sequence is *n*, then the structure of the number of *N* input frames S(N) is made from the *X* sequence as follows:

(4) S(N)={X[1],X[2],…,X[N]},

where

(5) $$X\left[i\right]=X[round\left(\frac{n-1}{N-1}\times \left(i-1\right)\right)].$$

**Figure 7:**
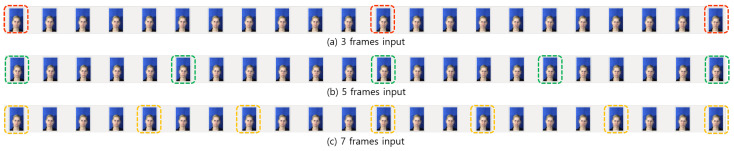
The example of the original structure of selecting input frames.

However, in this case, the first X[1] and the last X[N] images are always given as an input into each network. Additionally, middle part of the input can be overlapped. Then, the same information is overlapped into each network. As a result, the same spatial features are extracted. This is not good situation to learn the given input sequences. The example of the original structure of picking 3, 5, and 7 input frames is in Figure 7.

As in Figure 7, when *n* = 22, which means the sequence has 22 image frames, 3 frames of input are selected as *S*(3) = {*X*[0], *X*[11], *X*[21]}. In the case of 5 frames of input, *S*(5) is chosen as {*X*[0], *X*[5], *X*[11], *X*[16], *X*[21]}, and 7 frames of input sequence are constructed as *S*(7) = {*X*[0], *X*[4], *X*[7], *X*[11], *X*[14], *X*[18], *X*[21]}. All of them have the same images of *X*[0], *X*[11], and *X*[21] when constructing input sequences. In terms of information, the overlapped portion is not desirable to make reliable learning.

In order to solve this problem, we designed an input frame structure that can make a minimized overlapped between the generated input sequences. We extracted frames with regular intervals the same as the existing structure, but it made a different condition by making the start and end points different. The equation for the structure of the number of 3, 5, and 7 input frames S(N) from the original *X* sequence where the number of the sequence is *n* as follows:

(6) S(N)=X[1],X[2],…,X[N],

where

(7) $$X\left[i\right]=\left\{\begin{array}{cc} X[0+round\left(\frac{(n-1)-2}{3-1}\times \left(i-1\right))\right], & N\;=\;3\;\mathrm{input}\;\mathrm{frames}.\\ X[2+round\left(\frac{(n-1)-2}{5-1}\times \left(i-1\right))\right], & N\;=\;5\;\mathrm{input}\;\mathrm{frames}.\\ X[1+round\left(\frac{(n-1)-2}{7-1}\times \left(i-1\right))\right], & N\;=\;7\;\mathrm{input}\;\mathrm{frames}. \end{array}\right.$$

**Figure 8:**
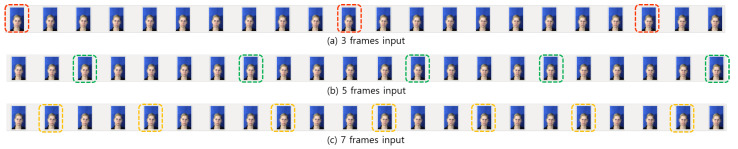
The example of selecting input frames through the minimum overlapped frame structure.

The start and end point of the seven input frames were set between the start and end points of the three and five input frames. In our example, seven frames was the largest number of the selected frames in a sequence with the number of *n*. If the start and the end point of seven input frames is shifted by one order from other input frames, the probability of overlap may be decreased. The example of the designed minimum overlapped frame structure, which selects three, five, and seven of input frames, is shown in Figure 8.

When *n* = 22, three frames of input have S(3) = {*X*[0], *X*[10], *X*[19]}. Five frames of the input sequence can be selected as *S*(5) = {*X*[2], *X*[7], *X*[12], *X*[16], *X*[21]}, and seven frames of input are constructed by *S*(7) = {*X*[1], *X*[4], *X*[8], *X*[11], *X*[14], *X*[17], *X*[20]}. None of the input images overlap as shown in Figure 8. The proposed structure can give more spatio-temporal information to extract features in the neural network. With the suggested three multi-depth network, full frames cannot be utilized. However, if we add one or two more different depth networks, then we can utilize almost-full frames with a larger frame rate for our FER task.

### 3.2. Data Preparation

#### 3.2.1. Data Augmentation

For FER, we needed enough datasets of human faces. However, most of the FER databases have been labeled with a well-controlled environment, and it needs a high-cost task. Therefore, there are not enough datasets for the experiment in most cases. When training through deep learning with insufficient datasets, the network can be easily overfitted. Therefore, most researchers use data augmentation to solve this overfitting problem.

**Figure 9:**
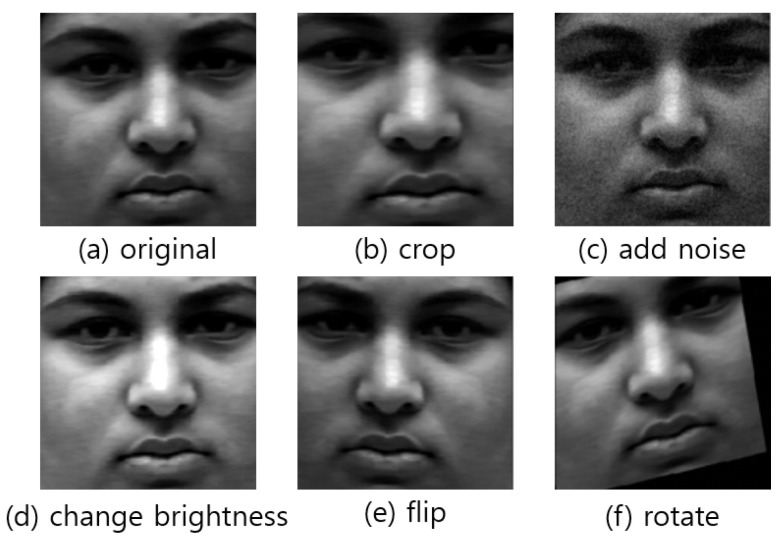
The example of data augmentation.

Data augmentation is largely divided into two types. The first method is to utilize some deep learning technologies such as autoencoder (AE) [22,55] or generative adversarial networks (GAN) [56]. Usually, autoencoder (AE) [22,55] with generative adversarial networks (GAN) [56] together could be used for input data augmentation. The second method is augmentation through image pre-processing like rotation, skewing, and scaling. Flipping horizontally is also effective in increasing the dataset. This is effective to increase the number of data while maintaining the geometric relationship between the eyes of the face image and important parts of the face such as the nose and mouth. Another method is to add noise to the image. This method includes salt and pepper noise, speckle noise, and Gaussian noise. In [57], the amount of the dataset was increased by 14 times through horizontal flipping and rotation. Figure 9 shows data augmentation using image pre-processing.

In this experiment, the second data augmentation method was used to increase the amount of the dataset. Table 1 shows the number of the original input dataset in each database. The CK+ database contains images labeled with “contempt,” but other databases do not have this label [58,59]. Therefore, we excluded sequences labeled with “contempt” to establish the same experimental conditions. For the MMI dataset [60], we separated frames for each emotion before making inputs.

In the case of the GEMEP-FERA database [61], the total number of the emotion class was 5. However, one of them was not “neutral” but had a label of “relief.” We changed the label of “relief” into “neutral.” In Table 1, the first row is an abbreviation for the emotion classes such as neutral, anger, disgust, fear, happiness, sadness, and surprise in that order. The AFEW database [62] has already been divided into training, validation, and test datasets. However, the test dataset had no annotation about the expression. Therefore, we used the provided train dataset for the train, and the validation dataset was used for the test stage.

The expression input data set was constructed in the following way. First, several frames were extracted from the input sequence through the minimum overlapped fame structure. If there was a separate sequence with the neutral label in the database, the neutral label dataset was also configured in the same way. However, in the case of a database where the neutral label was not specified, the neutral dataset was created through the first three frames of each sequence.

In the case of CK+ databases, there were no neutral labeled sequences. Therefore, a labeled emotion dataset was created through the minimum overlapped frame structure method, and an neutral dataset was created through the first three frames of each sequence. Because of this, datasets labeled with neutral existed in all sequences. Since each emotion-labeled dataset can only be created in a specific labeled sequence, the difference between the amount of neutral datasets and the other emotion datasets became large. In order to avoid the overfitting problem that can be caused by insufficient and biased distribution of the datasets, it was necessary to increase the emotion-labeled dataset.

Data augmentation was mainly performed to increase the amount of the emotion-labeled dataset so that the dataset was evenly distributed. For the created neutral expression dataset, each image was flipped horizontally to increase two times. For the other dataset, each image was flipped horizontally and rotated by {$-7.5^{\circ }$, $-5^{\circ }$, $-2.5^{\circ }$, $2.5^{\circ }$, $5^{\circ }$, and $7.5^{\circ }$}. Through this process, the emotion-labeled dataset increased 14 times. Table 2 shows the specific values of the increased datasets for the CK+, MMI, and GEMEP-FERA datasets. In particular, we augmented two times for the neutral dataset, which was created from all emotion-labeled sequences due to no neutral emotion in the MMI dataset. The ‘−‘ symbol in Table 2 means that the class does not exist in the GEMEP-FERA dataset.

The provided AFEW train dataset, which was used as a train and validation dataset in our experiment, was augmented four times. We flipped horizontally and rotated by {$-2.5^{\circ }$, $2.5^{\circ }$}. The provided AFEW validation dataset, which was used as a test dataset in our experiment, was augmented two times by flipping horizontally. The result of the augmented AFEW dataset is in the fifth and sixth rows of Table 2.

#### 3.2.2. Making Neutral Label of Dataset

**Figure 10:**
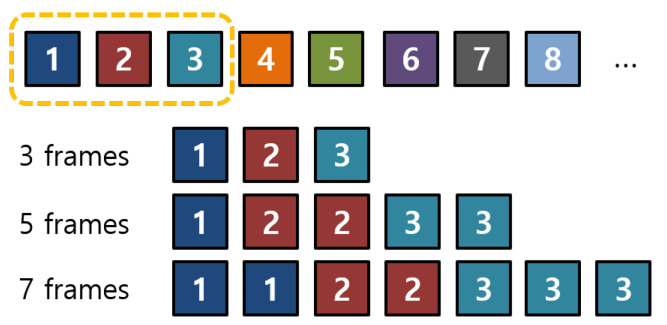
The method of creating a neutral dataset where the neutral label is not specified.

The CK+ database is composed of images to go from neutral to the peak of expression. Thus, the neutral sequence in the CK+ database is at the beginning of the video. To make three consecutive frames as inputs, the first three frames were assigned to the frames labeled as neutral. Input consisted by five frames was made by using the first frame once, the second frame twice, and the third frame twice among the first three consecutive frames. Input consisted by seven frames was created by using the first frame twice, the second frame twice, and the third frame three times among the first three consecutive frames. Figure 10 shows an example of a “neutral” label frame extracted from a sequence.

Unlike the CK+ database, the MMI database had an emotion flow, which was “neutral” to one of the peaks of expression and then to “neutral.” We judged that the peak of the emotion was in the middle of the video. Therefore, the dataset was created using only the half that was the first to middle sequence out of the total sequence. Then, it had the same emotion flow like in the CK+ database as the “neutral” emotion to the peak of one expression. The dataset for the neutral expression was made through the same method, which created the neutral dataset from the CK+ database.

On the other hand, the GEMEP-FERA database did not have a label for “neutral” but a label for “relief.” In order to match the conditions with other databases, we defined the “relief” as the “neutral” label.

### 3.3. 3D Convolutional Neural Network (3D CNN)

Spatial and temporal information was simultaneously captured using a 3D convolution and a 3D input dataset. Unlike the kernel used in 2D CNN, 3D CNN has a 3D cube-shaped convolution kernel, which has one more depth in the time axis. This preserves the time information of the input sequence and creates an output that forms the volume. Therefore, motion information can be obtained by connecting the feature map of the convolutional layer from multi-frames as input. Additionally, it considers adjacent pixels within the frame like the operation of 2D convolution at the same time. Therefore, spatial and temporal information can be simultaneously extracted through 3D convolution.

Shuiwang et al. [24] have explained 3D CNN mathematically. The value at position (x,y,z) on the *j*-th feature map in the *i*-th layer is given by:

(8) $$v_{ij}^{xyz}=tanh\left(b_{ij}+\sum_{m}\sum_{p=0}^{P_{i}-1}\sum_{q=0}^{Q_{i}-1}\sum_{r=0}^{R_{i}-1}w_{ijm}^{pqr}v_{(i-1)m}^{\left(x+p)(y+q)(z+r\right)}\right),$$

where (p,q) is the spatial dimension index, *r* is the temporal dimension index of the kernel, $w_{ijm}^{pqr}$ is the (p,q,r)-th value of the kernel connected to the *m*-th feature map in the previous layer, and $R_{i}$ is the size of the 3D kernel. tanh( ) assumed that activation function is the hyperbolic tangent, so other activation function can also be used.

**Figure 11:**
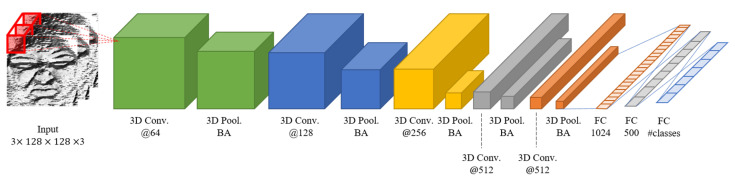
The architecture of 3D CNN from study [26], which has five 3D convolution layers.

In this study, we used a 3D CNN from study [26], which is called an “Appearance Network,” as a basic model to capture spatio-temporal information. Figure 11 shows the detailed configuration of the network.

First, the 3D convolutional layer extracts spatial and temporal features. All convolutional layers use a 5 × 5 × 3 kernel and a restricted linear unit (ReLU) activation function. In addition, 3D pooling is applied to reduce the number of parameters and cope with changes in the position of image elements. In this case, the pooling layer is max pooling that transfers only the maximum value of the volume area. After the maxpooling operation, the size of the feature map is reduced. Due to 3D pooling, dimension reduction on the time axis also occurs. The maximum value of 2 × 2 × 2 blocks is mapped to a single pixel of the output 3D feature map.

After max pooling layers, a batch normalization layer follows. Batch normalization is one of the ideas for preventing the disappearance or explosion of the gradient [63]. During deep learning training, if the hierarchy is deep and the number of epochs increases, the slope may explode or disappear. This problem arises because the scale of the parameters is different. This means the distribution of input to each layer or activation function of the network would be better to be controlled in the signal scale. To solve this problem, the input distribution needs to be normalized. However, this method is very complicated because the covariance matrix and the inverse matrix must be calculated. Instead, through batch normalization, the mean and standard deviation are obtained from each feature rather than the entire dataset, and they are normalized for each feature.

At the end of the network, emotions are classified as consecutive values through the softmax function. However, this classification module is not used in this study because we designed different joint fusion classifier based on the self-attention.

### 3.4. Joint Fusion Classifier Using Self-Attention

In this section, a joint fusion classifier is designed for a combination of multiple networks. This classifier serves to classify facial expressions based on various pieces of information by combining features extracted from each different input frame. In other words, it is possible to obtain more accurate results by supplementing the results of each network. Here, feature vector 1,…, *N* were extracted to make the final 3D features from each depth network in Figure 11. In this study, there were three features since we employed three depth networks. When we extended this up to the *N* depths network, we could obtain *N* number of features before the classification module.

Additionally, we employed a squeeze-and-excitation network (SENet) for self- attention [46]. For any given transformation $F_{tr}:X\to U$, $X\in R^{H^{\prime }\times W^{\prime }\times C^{\prime }}$, $U\in R^{H\times W\times C}$ (e.g., a convolution or a set of convolutions), we employed the squeeze-and-excitation (SE) block [46] to perform feature re-calibration as follows. In this structure, the features *U* are first passed through a squeeze operation, which aggregates the feature maps across spatial dimensions H×W to produce a channel descriptor. This descriptor embeds the global distribution of channel-wise feature responses, enabling information from the global receptive field of the network to be leveraged by its lower layers. This is followed by an excitation operation, in which sample-specific activation, learned for each channel by a self-gating mechanism based on channel dependence, govern the excitation of each channel. The feature maps *U* are then re-weighted to generate the output of the SE block, which can then be fed directly into subsequent layers.

**Figure 12:**
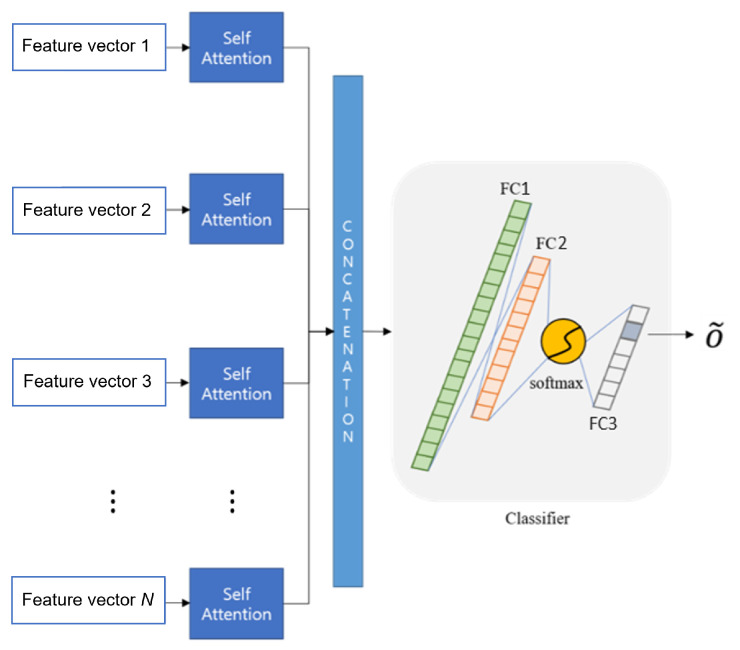
The architecture of the joint fusion classifier using self-attention.

Therefore, we could obtain emphasized and reinforced features through self-attention. Those features were concatenated in one-dimension and fed into the joint fusion classifier, which is depicted in Figure 12.

In Figure 12, a joint fusion classifier was composed as follows: fully connected (FC) layer one and fully connected (FC) layer two of each network use ReLU. Fully connected (FC) layer three uses the softmax as an activation function. Additionally, cross entropy was used as the loss function, and loss was reduced by using the Adam optimizer. This determined the final emotion and used the same training dataset for each network to use it.

As mentioned in the above, we designed a multi-depth network based on multi-rate feature fusion for efficient facial expression recognition. Additionally, we developed a new image normalization and different depth networks as frame rates to give more robustness for various datasets. We verified the robustness and effectiveness of the proposed algorithm through experiments.

## 4. Experimental Results and Discussion

This section introduces the experiment and its environment in detail. We present and analyze the performance through several experimental results. Additionally, we compare the proposed FER algorithm with other latest algorithms. To train this network, the Adam optimizer was used with the default parameter setting [64]. We implemented all methods on a GPU server with Intel i-7 CPU and GTX 1080 Ti 11G memory.

### 4.1. Ablation Study

#### 4.1.1. Performance of Image Normalization

**Figure 13:**
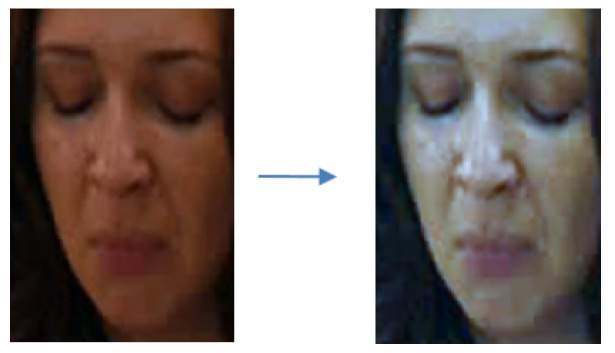
Examples of applying image normalization in AFEW dataset.

This experiment confirmed the better performance when the image was normalized as described in Section 3.1.1.3. In the AFEW dataset, most of the image sequences were not taken from the controlled environment but were the same as in real-life conditions. Therefore, the brightness of the images varied, even being too dark or too bright. By using image normalization, we could overcome such problems, and the result of using image normalization is shown in Figure 13.

The results of image normalization in CK+, MMI, GEMEP-FERA, and AFEW datasets is in Table 3. In MMI and GEMEP-FERA datasets, this method mostly showed a better result than not using image normalization. The bold letter in the Table 3 means a better or same accuracy than not using image normalization. In the CK+ database, the employed image normalization was 0.14% better on average. However, in MMI, GEMEP-FERA, and AFEW datasets, most of the results using image normalization showed better performances of 0.7%, 0.61%, and 0.23% on average.

#### 4.1.2. Correlation between Depth of the Network and Frame Rate of Input

This experiment was to find out the correlation between the depth of the network model and the number of the input frame. We took the experiment with applying and transforming the depth of the base model (5 layers) based on the 3D appearance network [26]. We gave three, five, and seven frames input into the 3D CNN with 5 (base model), 10, 15, 20, and 25 layers. As mentioned, we gave the depths of the models as 5 layers, 10 layers, 15 layers, 20 layers, and 25 layers to check on the relationship.

**Figure 14:**
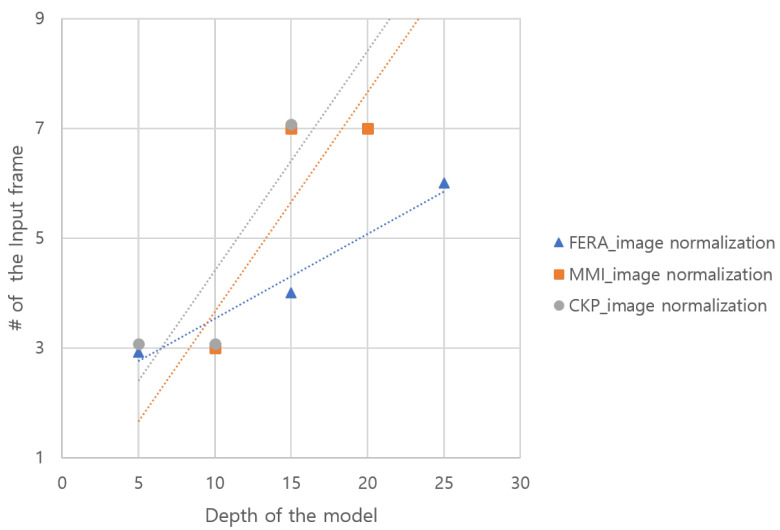
The graph of correlation between the depth of network and frame rate of the input.

We used CK+, MMI, and GEMEP-FERA datasets to deduce the relationship. The results of the experiment by combining each depth of the network and input frame rate are shown in Table 4. The bold face denotes the maximum accuracy for each network depth according to input frame rate. In Figure 14, it was converted into a graph to visually show the results of Table 4. The dotted lines indicate the trend line.The result shows that if the depth of the model and the frame rate of the input are proportional, then the accuracy is inclined to increase. This means the accuracy is higher as the depth of the model is large and the number of frames of the input increases. Additionally, as the depth of the model is shallow and the number of frames of the input is smaller, the accuracy tends to be high. We utilize this observation to design our multirate-based network model.

#### 4.1.3. Performance of the Minimum Overlapped Frame Structure

In this experiment, when creating the input dataset structure that is used in multiple networks, we verified that more various temporal information is helpful for learning. The minimum number of frames in the dataset was set to nine frames. Previously, the input dataset entering each network was determined as follows. If there is an arbitrary sequence of images, the total number of images is divided by equal intervals to obtain the required number of input frames. In this case, the beginning and end of three frames of input, five-frames of input, and seven frames of input always contained the same image. It means that the probability of overlapping the intermediate image was also high. In order to compensate for this problem, the method described in Section 3.1.2 was designed to create an input frame that does not overlap as much as possible. Because of the minimum overlapped frame structure, it was possible to give more various information when the network was learning.

Based on the correlation between the depth of the network and the frame rate of the input in Section 4.1.2, we fed 3 frames of input into the 3D CNN with 5 layers, 5 frames input into the 3D CNN with 10 layers, and 7 frames input into the 3D CNN with 15 layers. We obtained the feature from the networks without using image normalization and LBP feature extraction. Through Table 5, it can be seen that providing a variety of information to the network improves the performance in all of the databases. In the CK+, MMI, and GEMEP-FERA datasets, better performances of about 1.97%, 1.53%, and 0.46%, respectively, were shown. Moreover, the network using a minimum overlapped frame structure showed an improvement of 0.97% in the AFEW database.

#### 4.1.4. Performance of Self-Attention Module

We also fed 3 frames into the 3D CNN with 5 layers, 5 frames into the 3D CNN with 10 layers, and 7 frames into the 3D CNN with 15 layers using a minimum overlapped frame structure, and we did not use the image normalization and LBP feature extraction. When the features came out from each network, we reinforced the feature using the self attention. Then, we concatenated the reinforced features into one-dimension and fed them into the joint fusion classifier.

We checked whether the self-attention module reinforced the features or not by comparing between the concatenated feature without the self-attention module and the concatenated feature with the self-attention module. The result is shown in Table 6. We can see that the self-attention module reinforced the feature and improved the FER performance in most of the databases. In the CK+, MMI, and GEMEP-FERA databases, the proposed scheme showed about 0.21%, 0.91%, and 0.23% better performances, respectively. Additionally, in the AFEW database, it showed a 0.42% better performance with the self-attention module.

#### 4.1.5. Effectiveness of Multi-Depth Network Structure

To show the effectiveness of the proposed multi-depth network structure, we tested a single layer network, which was from [26], as shown in Figure 11. We set three frames as the input sequence. For obtaining the results, we used a 10-fold validation approach.

Table 7 summarizes the number of trainable parameters of the proposed three-depths network model. It was assumed that three frames were given as input. It is also showed only the layers with trainable parameters in the entire network. As shown in the table, the number of layers in the individual network increased to a multiple of fie according to the number of frames given as input, and the number of parameters increased significantly accordingly. The outputs of each network were finally combined into the last three FC-layers, with the total number of parameters including them reaching about 237 million. If we extend the proposed network with more depths, then the complexity will be further increased.

For the CK+ dataset, the proposed multi-depth network gave slightly better accuracy than the single network in Table 8. Additionally, we observed up to 7% of the accuracy in the MMI and GEMEP-FERA datasets. From these results, we can conclude that the proposed multi-depth network was effective for the facial expression recognition task.

### 4.2. Overall Accuracy Performance of the Proposed Scheme

In this section, we demonstrate that the proposed scheme shows competitive performance compared with the recent existing methods. Among various techniques for facial expression recognition, we compared with spatio-temporal network approaches or hybrid network approaches. Table 9 shows input construction and model setting of the recent existing methods, which were compared with the proposed method.

For experiments, we used three datasets: the CK+, MMI, and GEMEP-FERA datasets. The number of image sequences in each dataset was listed in Table 2. We used 3 frames, 5 frames, and 7 frames as input, and the multi-depth network was composed of 5 layers, 10 layers, and 15 layers. We used self-attention to reinforce the features, which came from each network and fed into the joint fusion classifier.

The results from the 10-times trial on the CK+ dataset are in Table 10. “Without Pre-processing” means that we did not use the image normalization, the LBP feature extraction, the minimum overlapped frame structure, and the self-attention module. In contrast, “With Pre-processing” means that we used all proposed image pre-processing methods, including the minimum overlapped frame structure and the self-attention module. The average accuracy was shown as the bold face in each processing. For the CK+ dataset, the network performance of “Without Pre-processing” showed better results—about 1.11% on average. This CK+ dataset is a very static one. However, the proposed scheme was based on several video frames to extract more temporal information. This means that the proposed algorithm works well for more dynamic video sequences.

The accuracy comparisons of each method using the CK+ database is shown in Table 11. For the CK+ database, the proposed scheme which was denoted as the bold face, did not get the best result compared with some existing methods [26,68,69,70].

The results from the 10-times trial on the MMI dataset are in Table 12. The proposed scheme showed a better result by about 4.79% on average (as the bold face) than “Without Preprocessing.” The comparison of experimental results showed the outperformed results for the MMI dataset in Table 13. Here, the bold face denotes the performance of the proposed scheme.

Additionally, the proposed method outperformed in the GEMEP-FERA dataset. The result from the 10-times trial on the GEMEP-FERA dataset is displayed in Table 14. The network performance of “With Pre-processing” showed better results of about 0.64% in average (as the bold face) than “Without Pre-processing.” Table 15 shows the comparison of experimental results in the GEMEP-FERA dataset. The proposed scheme (the bold face in average) achieved an improvement of 8%, at least compared to the recent methods.

The proposed method showed a little weak performance on the CK+ dataset. However, in the MMI and GEMEP-FERA datasets, it showed the highest performance. According to the results of the CK+, MMI, and GEMEP-FERA datasets, the proposed model showed better performance in the more complex dataset.

In the AFEW dataset, the result is shown in Table 16. The AFEW dataset is well known as data capture in a very wild environment. The network performance of “With Pre-processing” showed a result that was about 3.32% better than “Without Pre-processing” by using only video data. From this result, we can expect that the proposed scheme can improve the recognition accuracy of the facial expression in real environments.

For the processing time of the proposed scheme, the inference time was measured. This inference time contained the consumed time of the data pre-processing, the construction of frame structures, and the prediction for the final decision. When testing the proposed multi-depth network (three layers and three, five, and seven frames of input), the inference time was measured by about 102.0 ms on our GPU server with an Intel i7 CPU and GTX 1080 Ti 11G memory. In terms of the frame processing rate, a value of 9.8 frames per second (FPS) was obtained. When we used a single0layer network with an input of three frames, as shown in Figure 11, 49.3 ms was measured due to a very small network structure.

## 5. Conclusions

We proposed a robust facial expression recognition algorithm on the variation of datasets and different facial expression acquisition conditions. The proposed scheme extracted various features by combining several networks based on external features and classified them by putting them in a joint fusion classifier. This network simultaneously extracted spatial and temporal features using 3D CNN to overcome the problem of the existing 2D CNN model trained only with spatial features. In addition, in order to obtain a various features, we designed a multi-depth network structure by multiple input frames which were the least overlapped and composed of LBP features. The features extracted from each network were reinforced through the self-attention module. Then, these were combined and fed into the joint fusion network to newly learn and classify the emotions.

Through experiments, we found the correlation between the number of input frames and the depth of the network. When the number of frames increases, the network depth increases. When the number of frames decreases, the shallower the network depth, which showed the better performance. Through comparative analysis, we proved that the proposed multirate feature fusion scheme could achieve more accurate results than the state-of-the-art methods. The performance of the proposed model enhanced by 96.23%, 96.69%, and 99.79% the average accuracy of the CK +, MMI, and GEMEP-FERA datasets, respectively. Additionally, a 31.02% accuracy was achieved in the AFEW dataset through the features enhanced by the self-attention module and the proposed multi-depth network structure.

## Figures and Tables

**Table 1:** The number of the original input datasets.

	Neu.	Ang.	Dis.	Fea.	Hap.	Sad.	Sur.	Total
CK+	0	45	56	24	69	28	78	300
MMI	0	33	32	28	42	32	41	208
FERA	31	32	−	30	31	31	−	155
AFEW (Train + Val.)	143	146	77	80	156	119	74	795
AFEW (Test)	63	61	39	41	60	54	43	361

**Table 2:** The number of the augmented input datasets.

	Neu.	Ang.	Dis.	Fea.	Hap.	Sad.	Sur.	Total
CK+	600	630	784	336	966	392	1092	4800
MMI	416	462	448	392	588	448	574	3328
FERA	434	448	−	420	434	434	−	2170
AFEW (Train + Val.)	572	584	308	320	624	476	296	3180
AFEW (Test)	126	122	78	82	120	108	86	722

**Table 3:** The results of image normalization.

Datasets	Input Frames	Depth of Network	Image Normalization	
			Not Used	Used
CK+	3	5	98.65	98.02
		10	98.02	**98.33**
		15	97.92	97.81
MMI	3	5	95.58	**96.19**
		10	94.97	**97.1**
		15	94.40	93.75
FERA	3	5	98.85	**100**
		10	99.77	**99.77**
		15	99.31	**100**
AFEW	3	5	28.32	28.95
		10	26.59	**27.49**
		15	23.41	**23.89**

**Table 4:** The results of correlation between the depth of network and frame rate of the input.

Datasets	Depth of Network	Input Frames	Image Normalization
CK+	5	3	**98.45**
		5	98.34
		7	98.23
	10	3	**98.89**
		5	97.90
		7	98.31
	15	3	97.90
		5	97.23
		7	**98.45**
MMI	10	3	**96.65**
		5	95.88
		7	93.75
	15	3	89.84
		5	92.99
		7	**94.67**
	20	3	90.85
		5	89.94
		7	**92.84**
FERA	5	3	**100**
		5	99.54
		7	99.54
	15	3	**100**
		5	**100**
		7	99.77
	25	3	98.85
		5	**99.77**
		7	**99.77**

**Table 5:** The results of using minimum overlapped frame structure.

Datasets	Minimum Overlapped Frame Structure	
	Not Used	Used
CK+	96.88	98.85
MMI	89.48	91.01
FERA	99.31	99.77
AFEW	27.70	28.67

**Table 6:** The results of the performance using self-attention.

Datasets	Self-Attention	
	Not Used	Used
CK+	98.85	99.06
MMI	91.01	91.92
FERA	99.77	100.00
AFEW	28.67	29.09

**Table 7:** Summary of trainable parameters of the proposed multi-depth network (*input shape = (3, 128, 128, 1)*).

	Depth Network 1		Depth Network 2		Depth Network 3	
**Structure**	**Layers**	**Params**	**Layers**	**Params**	**Layers**	**Params**
						14,720
						307,520
				14,720		307,520
				307,520		615,040
				615,040		1,229,440
		14,720		1,229,440		1,229,440
Conv3d		615,040		2,458,880		2,458,880
+MaxPool	5	2,458,880	10	4,916,480	15	4,916,480
+BatchNorm		9,832,960		9,832,960		4,916,480
		19,663,360		19,663,360		9,832,960
				19,663,360		19,663,360
				19,663,360		19,663,360
						19,663,360
						19,663,360
						19,663,360
Self-attention	1	32,065	1	32,065	1	32,065
	1,537,024					
Fully connected	512,500					
	3,507					
**Total params**	237,244,586					

**Table 8:** The performance results of the proposed network (multi-depth network) and single network (%).

Datasets	CK+	MMI	GEMEP-FERA
Single Network	96.11	92.52	93.38
Proposed (Multi-depth network)	96.23	96.69	99.79

**Table 9:** Analysis of the recent existing methods for performance comparison.

Method	Datasets	Input Construction	Models
3DIR [65]	CK+, MMI, GEMEP-FERA	Multiple frames, facial landmarks	3D CNN, LSTM, Inception-ResNet
STCNN-CRF [66]	CK+, MMI, GEMEP-FERA	Multiple frames	2D CNN, CRF, Inception-ResNet
CNN-CTSLSTM [67]	CK+, MMI, AFEW	Multiple frames, facial landmarks	VGG-CTSLSTM, LEMHI-VGG
DDL [68]	CK+, MMI	Multiple frames	DFEM, DDM
DJSTN [26]	CK+, MMI, GEMEP-FERA	Multiple frames, facial landmarks	Hybrid network (App., Geo.)
FDRL [69]	CK+, MMI	Multiple frames	ResNet-18, FDN, FRN
MC-DCN [70]	CK+, MMI, GEMEP-FERA	Multiple frames	Hybrid network (C3Ds)

**Table 10:** Overall accuracy and improvement on the CK+ dataset (%).

	without Preprocessing	with Preprocessing
1	96.88	95.31
2	97.08	95.52
3	97.60	95.94
4	97.71	96.15
5	97.60	96.25
6	97.81	96.77
7	97.71	97.40
8	97.08	95.63
9	96.88	96.67
10	97.08	96.67
**Average**	**97.33**	**96.23**

**Table 11:** Comparison results of accuracy in the CK+ dataset (%).

Methods	Accuracy
3DIR [65]	93.21
STCNN-CRF [66]	93.04
CNN-CTSLSTM [67]	93.90
DDL [68]	99.16
DJSTN [26]	99.21
FDRL [69]	99.54
MC-DCN [70]	95.50
**Proposed Scheme**	**96.23**

**Table 12:** Overall accuracy and improvement on the MMI dataset (%).

	without Preprocessing	with Preprocessing
1	89.48	97.87
2	92.07	98.02
3	92.23	95.27
4	92.53	96.95
5	93.29	97.26
6	91.31	95.12
7	92.53	95.43
8	89.18	95.88
9	92.99	97.25
10	93.45	94.35
**Average**	**91.91**	**96.69**

**Table 13:** Comparison results of accuracy in the MMI dataset (%).

Methods	Accuracy
3DIR [65]	77.50
STCNN-CRF [66]	68.51
CNN-CTSLSTM [67]	78.4
DDL [68]	83.67
DJSTN [26]	87.88
FDRL [69]	85.23
MC-DCN [70]	78.6
**Proposed Scheme**	**96.69**

**Table 14:** Overall accuracy and improvement on the GEMEP-FERA dataset (%).

	without Preprocessing	with Preprocessing
1	99.31	100.00
2	99.77	100.00
3	98.62	99.54
4	99.54	99.77
5	99.54	100.00
6	98.16	99.77
7	97.24	99.54
8	99.54	100.00
9	99.77	99.54
10	100.00	99.77
**Average**	**99.15**	**99.79**

**Table 15:** Comparison results of accuracy in the GEMEP-FERA dataset (%).

Methods	Accuracy
3DIR [65]	77.42
STCNN-CRF [66]	66.66
DJSTN [26]	91.83
MC-DCN [70]	78.3
**Proposed Scheme**	**99.79**

**Table 16:** Overall accuracy and improvement on the AFEW dataset (%).

	without Preprocessing	with Preprocessing
Accuracy	27.70	**31.02**

## Data Availability

All sources and data can be found at https://github.com/smu-ivpl/MultiRateFeatureFusion_FER (accessed on 18 October 2021).
